# Assessing the Assisted Six-Minute Cycling Test as a Measure of Endurance in Non-Ambulatory Patients with Spinal Muscular Atrophy (SMA)

**DOI:** 10.3390/jcm12247582

**Published:** 2023-12-08

**Authors:** Whitney J. Tang, Bo Gu, Samuel Montalvo, Sally Dunaway Young, Dana M. Parker, Constance de Monts, Paxton Ataide, Noirin Ni Ghiollagain, Matthew T. Wheeler, Carolina Tesi Rocha, Jeffrey W. Christle, Zihuai He, John W. Day, Tina Duong

**Affiliations:** 1 Department of Neurology and Clinical Neurosciences, Stanford University, Palo Alto, CA 94305, USA; whitneyt@stanford.edu (W.J.T.); sdy@stanford.edu (S.D.Y.); ctesiroc@stanford.edu (C.T.R.); zihuai@stanford.edu (Z.H.); jwday@stanford.edu (J.W.D.); 2 Department of Medicine, Division of Cardiovascular Medicine, Stanford University, Palo Alto, CA 94305, USA; smontal@stanford.edu (S.M.); christle@stanford.edu (J.W.C.)

**Keywords:** exercise, outcome measure, function, upper extremity, fatigability, nusinersen, risdiplam

## Abstract

Assessing endurance in non-ambulatory individuals with Spinal Muscular Atrophy (SMA) has been challenging due to limited evaluation tools. The Assisted 6-Minute Cycling Test (A6MCT) is an upper limb ergometer assessment used in other neurologic disorders to measure endurance. To study the performance of the A6MCT in the non-ambulatory SMA population, prospective data was collected on 38 individuals with SMA (13 sitters; 25 non-sitters), aged 5 to 74 years (mean = 30.3; SD = 14.1). The clinical measures used were A6MCT, Revised Upper Limb Module (RULM), Adapted Test of Neuromuscular Disorders (ATEND), and Egen Klassifikation Scale 2 (EK2). Perceived fatigue was assessed using the Fatigue Severity Scale (FSS), and effort was assessed using the Rate of Perceived Exertion (RPE). Data were analyzed for: (1) Feasibility, (2) Clinical discrimination, and (3) Associations between A6MCT with clinical characteristics and outcomes. Results showed the A6MCT was feasible for 95% of the tested subjects, discriminated between functional groups (*p* = 0.0086), and was significantly associated with results obtained from RULM, ATEND, EK2, and Brooke (*p* < 0.0001; *p* = 0.029; *p* < 0.001; *p* = 0.005). These findings indicate the A6MCT’s potential to evaluate muscular endurance in non-ambulatory SMA individuals, complementing clinician-rated assessments. Nevertheless, further validation with a larger dataset is needed for broader application.

## 1. Introduction

### 1.1. SMA: Overview of Clinical Outcome Assessments

Spinal Muscular Atrophy (SMA) is an autosomal recessive disorder characterized by motor neuron dysfunction and degeneration, which lead to progressive muscle atrophy, weakness, and early mortality [1,2]. Recent use of Disease Modifying Therapies (DMTs) has improved individual clinical courses, with earlier treatment predicting greater efficacy and outcomes [3,4,5]. However, a large majority of individuals continue to live with symptoms that impact activities of daily life, particularly those requiring sustained endurance. 

Motor Clinical Outcome Assessments (COAs) currently used for SMA include clinician-rated scales to assess functional abilities, such as the Hammersmith Functional Motor Scale Expanded (HFMSE), the 32-item Motor Function Measure (MFM32), and the Revised Upper Limb Module (RULM) [6,7,8,9,10,11,12,13]. These tests do not assess sustained activity, and variability in scores can reflect fatigability associated with administration time and the frequency of assessments. Potential floor and ceiling effects of these COAs impact the ability to measure change in function, as evidenced by a recent longitudinal study showing floor effects (<10 points on RULM, <5 points on HFMSE) and ceiling effects (>35 points on RULM and >60 on HFMSE) in adults with SMA [14,15,16]. SMA functional composite scores have been designed to broaden the psychometric limitations of singular tests [17]. However, the amalgamation of these assessments still leaves a gap in measurements of endurance capacity in non-ambulatory individuals. 

### 1.2. Current Evaluations for Endurance Capacity in SMA

Muscular endurance is important to measure in the SMA population as reduced endurance is a common yet under-assessed symptom that interferes with daily living [18]. Endurance can be defined as the prolonged maintenance of a constant or self-regulated power, velocity, or force level [19,20,21,22]. Endurance can be further broken down into muscular endurance, which is a muscle’s ability to continuously perform successive exertions or repetitions against a submaximal load, and aerobic endurance, which is the ability of the heart and lungs to fuel the body with oxygen [23,24,25]. This study will focus on muscular endurance.

For ambulatory individuals with SMA, the 6 Minute Walk Test (6MWT) is currently used as the validated gold standard for evaluating submaximal functional capacity (i.e., muscular endurance) [26]. The test is feasible, validated with existing motor outcomes, and responsive to treatment over time in the pediatric population [26,27]. However, there is no comparably accepted measure for non-ambulatory individuals. 

The Endurance Shuttle Box and Block Test (ESBBT) and the Endurance Shuttle Nine Hole Peg Test (ES9HPT) measure performance fatigability and are the first validated tests for proximal arm function in SMA [28]. Additionally, the Endurance Shuttle Tests have shown responsiveness in the SPACE trial [29,30]. However, these tests require a degree of distal motor coordination and strength, which is problematic for some of the weaker non-sitter population, as well as time, given that the test may range from a few minutes to 20 min [29]. 

In this study, the Assisted 6-Minute Cycling Test (A6MCT) was used to measure sustained muscular endurance during a 6-min interval with total revolutions as the primary metric of performance.

### 1.3. Assisted 6-Minute Cycling Test Use in Other Populations

The A6MCT serves as an effective ergometer for the assessment of endurance in non-ambulatory populations [31]. In an adult population with numerous neuromuscular diseases (e.g., Duchenne Muscular Dystrophy, Limb Girdle), the affected population consistently cycles fewer revolutions when compared to healthy controls [32]. Additionally, the A6MCT has been proven sufficiently sensitive in detecting differences between disease populations and progression [32]. Previously published research methodology on the A6MCT utilizes maximal-effort cues but appears to elicit sub-capacity effort from patients with NMD [31]. This is corroborated by other studies that confirm A6MCT distance/total revolutions is a validated measure of endurance for submaximal capacity for the NMD population [32,33,34]. Studies noted maximal-effort verbal cues were reliable indicators of consistency in effort throughout the 6 min. 

The A6MCT was also utilized in a crossover study as both an exercise intervention and a means of measuring endurance through total revolutions in Duchenne Muscular Dystrophy patients. The results revealed the maintenance of function after 24 weeks, in contrast to a significant decline observed in the control group as measured by MFM32 scores. The A6MCT and 9-hole peg test showed no significant changes, although the authors did specify that the training regimen was not designed to improve endurance [35].

### 1.4. Study Objective

To evaluate the applicability of the A6MCT in non-ambulatory individuals with SMA, our first objective was to assess its ability to capture muscular endurance measured by the total number of revolutions completed in 6 min. Our second objective was to establish convergent validity with existing clinical outcome assessments (COAs), including upper extremity (RULM) and wheelchair-based motor function outcomes (Adapted Test of Neuromuscular Disorders (ATEND)), as well as patient-reported outcomes (Egen Klassification Scale (EK2)), and perceptions of fatigue (Fatigue Severity Score (FSS)). Finally, we sought to evaluate the relationship to change over time. Our exploratory objective was to evaluate the feasibility of assessing fatigability comparing the first to the last minute of the A6MCT. 

## 2. Materials and Methods

### 2.1. Inclusion/Exclusion

Individuals were recruited from the Neuromuscular Clinics at Stanford University Health Care and Lucille Packard Children’s Hospital. Inclusion criteria were the following: genetic diagnosis of SMA, inability to perform 6MWT, ability to follow instructions and complete A6MCT. For this study, we defined a non-sitter as an individual unable to sit without upper limb support for 3 s (item 1 HFSME). We broadened the standard definition for the sitter group to include those who can only stand or weak walkers who cannot complete the 6MWT. Exclusion criteria included those who were too weak to keep their hands on A6MCT handlebars for the entirety of the 6 min even with assistance of gloves, individuals with severe upper extremity contractures that limited performance, or those with a history of cardiovascular disease that increased risk of strenuous exercise. Collected variables included demographics, vital signs, routine clinical measures, motor function outcomes, and patient-reported outcomes (Table 1). Assessments were performed in conjunction with routine clinical follow-up visits, making longitudinal timepoints variable. 

This study was approved by the Stanford University Internal Review Board (#54078) and all participants provided written informed consent and/or assent prior to study procedures. 

### 2.2. Performance Outcome (PerfO) of Endurance Using the A6MCT

**Figure 1:**
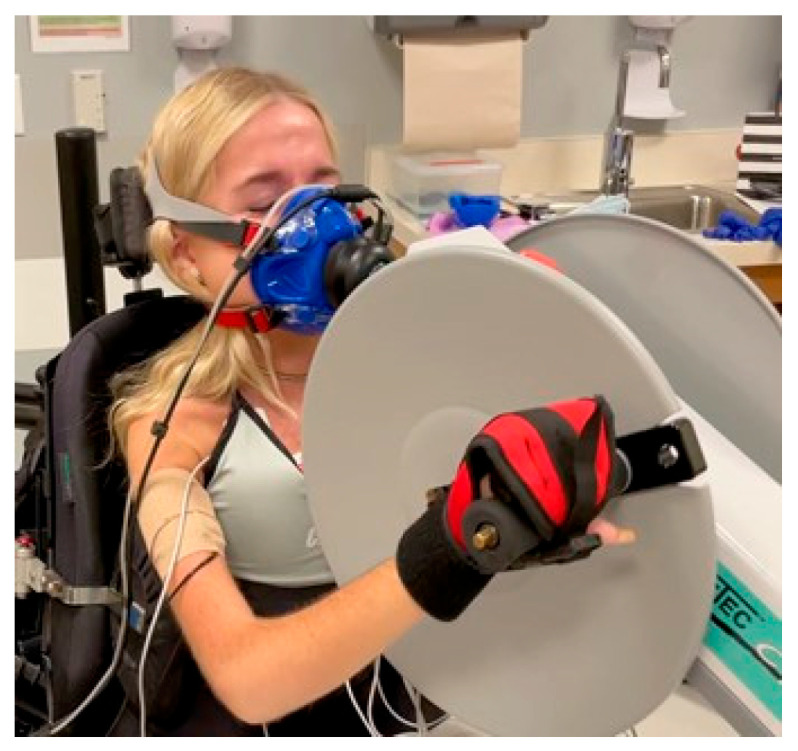
A6MCT Equipment Setup.

The A6MCT was performed using the Active Passive Trainer AP5 (Tzora Active Systems Ltd., Beachwood, OH, USA). The trainer was placed on an adjustable high-low table and the table’s height was adjusted for optimal performance based on previously published methodology [31]. Given the range of upper limb contractures and the presence of scoliosis, the bicycle was configured to achieve an optimal ergonomic setup to ensure unrestricted arm movement. In this process, we removed armrests and other supporting devices from the wheelchairs. If an individual was unable to maintain adequate grip for the A6MCT a hand glove was used to maintain grip. Outcomes from Near Infrared Spectroscopy (NIRS) and Cardiopulmonary Exercise Testing (CPET), such as VO2 max, watts, and heartrate, were collected simultaneously. We have chosen not to include findings in this initial analysis, as our primary aim is to concentrate on assessing feasibility and convergent validity. A picture of the equipment setup is included for reference (Figure 1). 

In accordance with previously established methodology, fixed motor assistance was used (passive mode 1, no-load speed 7 RPM) instead of resistance, which produced a baseline of approximately 52 cycles in 6 min for all participants. Participants were instructed to cycle as hard as possible to their maximal capacity for six minutes. Clinical evaluators provided consistent verbal motivation throughout the full six minutes. Testing was followed by two minutes of recovery. 

The primary variable evaluated in the A6MCT was distance, which is represented by the cumulative revolutions an individual completed at the end of 6 min, providing insight into patient endurance. Percent fatigue was used as an exploratory measure to assess ability to detect performance fatigability similar to 6MWT in stronger SMA populations [36]. Percent fatigue was determined as the difference in the revolutions cycled during the first and last minute expressed as a percent change, where a positive value represents fatigability. Other variables included revolutions per minute and effort represented by RPE. 

### 2.3. Clinical Assessments of Motor Function (RULM, ATEND)

Individuals were additionally assessed using SMA disease-specific motor function outcome measures based on functional ability. The RULM was performed for all individuals, as this is a validated outcome measure for upper arm strength in SMA and part of the minimal dataset predefined for collection as part of our clinical procedures [8]. Individuals also completed the Adapted Test for Neuromuscular Disease (ATEND), a novel motor function measure designed specifically for those who are in a wheelchair with neuromuscular disease [37]. Higher scores represent higher functionality. 

### 2.4. Perceived Fatigue and Effort (FSS, OMNI RPE)

Perceived fatigue was assessed with the Fatigue Severity Scale (FSS), a validated PRO in SMA for determining the impact of an individual’s recalled fatigue experience in the past week [38,39]. FSS assesses an overall exhaustion levels in conjunction with completing various activities and scores of >4 indicate “abnormal fatigue”, while > 5 indicate “severe fatigue”. Both FSS total score and FSS mean score were calculated, mean score reflecting the average of all items except 1 and 2. This item exclusion has been shown to increase the validity and reliability of the FSS mean score, making it a more sensitive method for measuring changes in fatigue [40]. 

Perceived effort related to task performance was measured by individuals reporting their Rate of Perceived Exertion (RPE) at the beginning of minute 1 and the end of minute 6 [38]. RPE is scored on a scale of 1–10. The RPE at minute 6 was used to characterize effort in our population.

### 2.5. Patient-Reported Outcome (EK2)

The final PRO assessed alongside the A6MCT was the Egen Klassifikation Scale Version 2 (EK2). This PRO measures an individual’s perceived motor function for different daily activities, which has an inverse relationship to the survey’s total score (lower score indicates better function) [41,42,43]. The scale has been validated in both DMD and SMA [43,44].

### 2.6. Statistical Analysis

Feasibility was determined by calculating the percentage of individuals who were able to perform the test. Test feasibility was defined as the percentage of individuals who successfully completed the A6MCT. Muscular endurance and performance fatigability on the A6MCT are expressed by continuous measures (number of revolutions, percent fatigue) and an ordinal scale (RPE).

Descriptive statistics (frequency with proportions for categorical/ordinal variables, means, medians, standard deviations, and ranges for continuous variables) were reported by demographic, clinical characteristics, and outcomes of interest.

Both Kolmogorov-Smirnov and Shapiro-Wilk tests were conducted to assess the normality of outcome variables. For outcomes that had significantly non-normal distributions, we implemented bootstrapping with 1000 replicates to randomly sample patients with replacements, which allowed us to make valid inference in finite sample scenarios. Specifically, bootstrapping on residuals was applied in both linear regression models and linear mixed models.

Convergent validity, which is defined by the COSMIN checklist as “the degree to which the scores of an instrument are consistent with predefined hypotheses regarding relationships to scores of other instruments”, was assessed using analyses at each patient’s baseline visit [45]. Baseline analysis assessed associations between A6MCT and clinical and patient-reported outcomes (including clinical characteristics and secondary outcomes), using the median values to account for the non-normal distribution. Simple linear regressions between A6MCT and the clinical measures were fitted on the overall sample by function groups. Multiple linear regressions between A6MCT and the clinical outcomes were performed and adjusted for the following covariates: age, functional status, SMN2 copy number, disease duration, and cumulative treatment duration. The modification effect of functional groups was assessed by introducing an interaction between function status and secondary outcomes (RULM, ATEND, EK2, FSS and RPE) in multiple regressions. We observed that both ATEND and Brooke significantly influenced the total revolutions on A6MCT, while performance fatigability was significantly associated with EK2. 

Longitudinal association between A6MCT and time from baseline (y) were analyzed using a series of linear mixed models (LMMs). For each LMM, time, along with age, functional status, SMN2 copy number, disease duration, and cumulative treatment duration were included as fixed factors. Each participant was entered as a random factor (random intercept only) within each LMM. The modification effect of time was assessed by introducing an interaction between time from baseline and secondary outcomes (% fatigue, RULM, ATEND, EK2, FSS and PRE) in multiple regressions. The best LMM was selected based on the Akaike Information Criterion (AIC), with lower values indicating a better-fitting model. Subsequently, model diagnostics were performed by examining residual plots and quantile-quantile (Q-Q) plots for each model. Significance was set a priori at *p* < 0.05.

## 3. Results

### 3.1. Demographics

#### 3.1.1. Clinical Characteristics

The clinical characteristics of all who were able to successfully complete the A6MCT are listed below in Table 2. It is worth noting that the sample size was largely non-sitters (65.8%) with an even split between SMA Type 2 and Type 3 (50.0%, 50.0%). While many individuals utilized the gloves due to grip weakness (34.2%), only a subset (7.9%) could not power the cycle above the natural assistance level (i.e., total revolutions did not exceed 52 in their baseline evaluation). 

When considering the feasibility of the A6MCT in SMA, only two individuals were not able to complete the test due to severe upper limb contractures preventing arm clearance to perform at least one revolution on the A6MCT, resulting in 95% of patients completing the test. As we did not obtain completed measurements for these patients, they are not included in the table below or in the analysis. Both patients were male, Type 2, and non-sitters.

#### 3.1.2. Descriptive Statistics of A6MCT Primary and Secondary Variables

A full breakdown of primary and secondary outcomes at baseline is listed below in Table 3.

**Figure 2:**
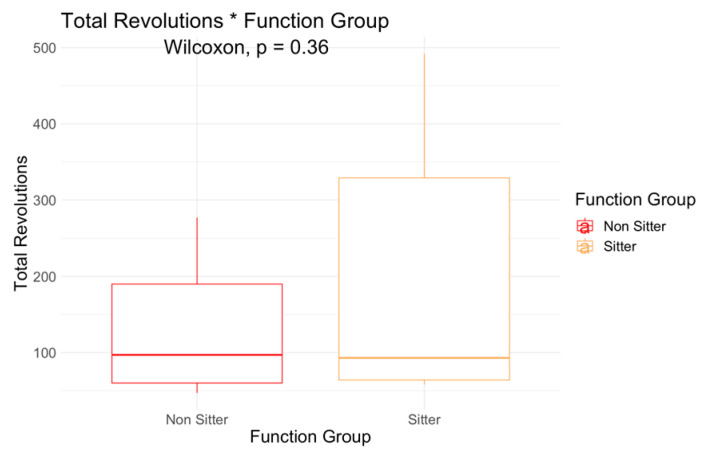
Total Revolutions Difference among Functional Groups.

**Figure 3:**
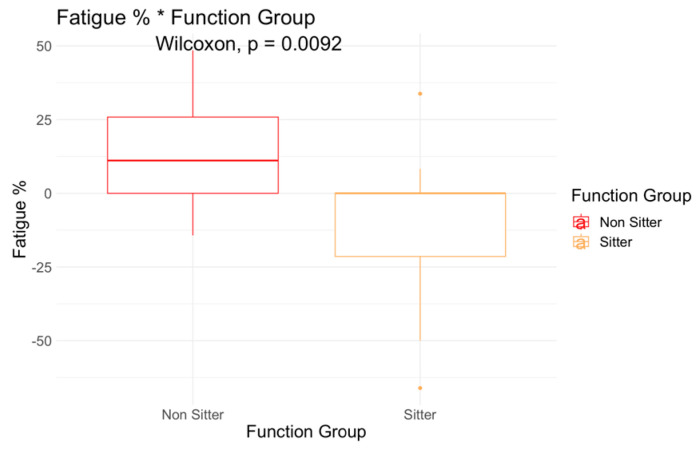
Percent Fatigue Difference among Functional Groups.

Total revolutions was higher in the sitters (204, SD 180) when compared to non-sitters (129, SD 77), but the difference was not significant (Figure 2). However, a Wilcoxon test comparison indicated a significant mean difference in performance fatigability between functional groups (*p* = 0.0092, Figure 3). Further, performance fatigability was positive in non-sitters (13.9, SD 19.3) indicating higher fatigability, but negative in sitters (−10.9, SD 27). Non-sitters scored lower in motor function measures, and reported more perceived fatigue (FSS), work effort (RPE), and performance fatigability (percent fatigue) when compared to sitters. 

### 3.2. A6MCT Associations to Clinical Characteristics and Secondary Outcomes

**Figure 4:**
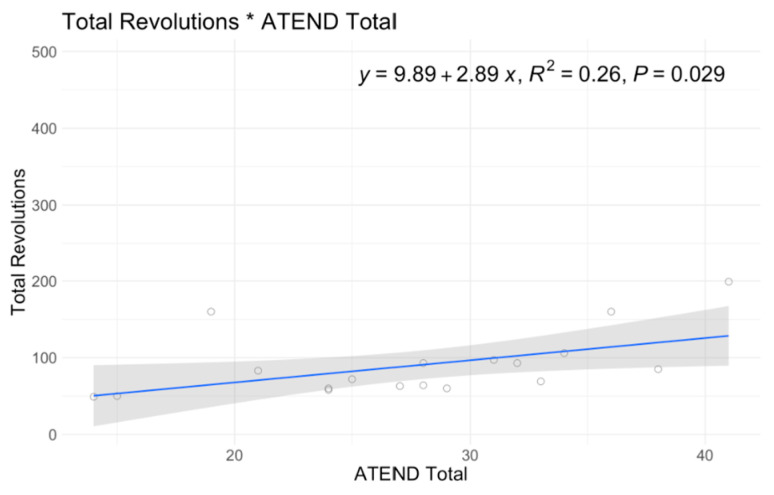
A6MCT Association with ATEND.

**Figure 5:**
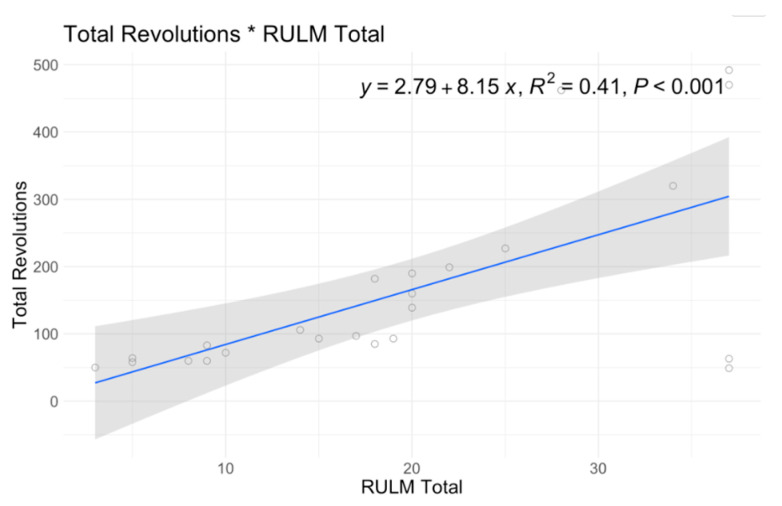
A6MCT Association with RULM.

**Figure 6:**
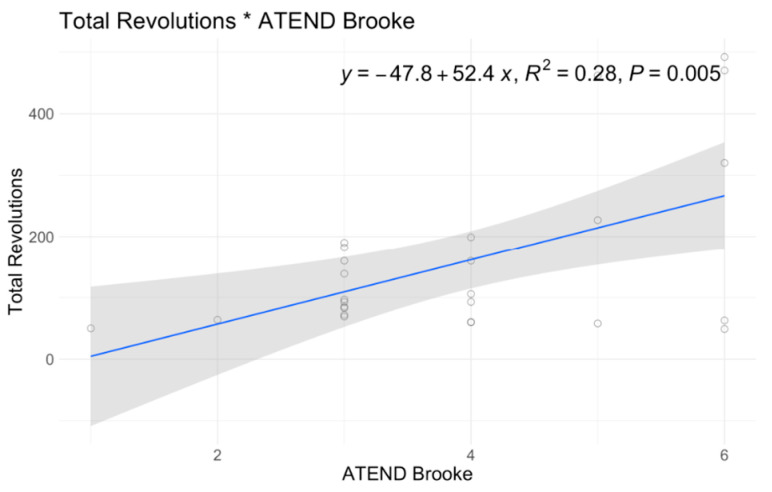
A6MCT Association with Brooke.

**Figure 7:**
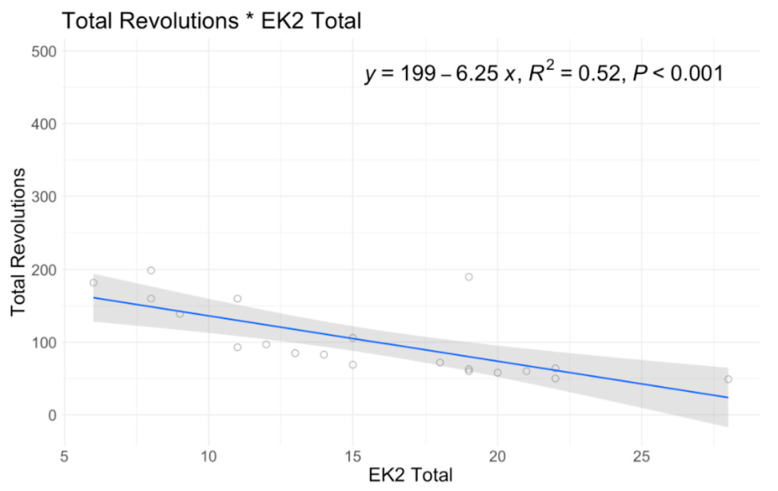
A6MCT Association with EK2.

In single variable associations, total revolutions was significantly correlated with RULM (*p* < 0.001), ATEND (*p* = 0.029), EK2 (*p* < 0.001), and Brooke Score (*p* = 0.005) (Table 4). Higher RULM and ATEND scores were significantly associated with more revolutions (r = 8.15; r = 2.89) (Figure 4 and Figure 5). Individuals with higher Brooke Scores (better function) achieved higher revolutions as well (r = 52.4) (Figure 6), while higher EK2 scores correlated significantly with 6.25 fewer revolutions per point change (Figure 7). Performance fatigability was found to have a minimal and statistically insignificant correlation with the Fatigue Severity Scale (FSS) (*p* = 0.365, r = −0.21) (Table 4).

ATEND score was significantly associated with a total revolution of 3.18 (*p* = 0.022). When sitters and non-sitters were evaluated as distinct cohorts, specific outcome measures revealed notable differences. Non-sitters demonstrated a significant positive correlation between total revolutions and ATEND (*p* = 0.025), unlike sitters, where each point increase was associated with a 3.91 increase in revolutions. For non-sitters, higher EK2 scores (less function) were significantly associated with a decrease in total revolutions 6.31 (*p* = 0.002) while sitters showed a decrease of 3.82 (*p* < 0.001). 

### 3.3. Longitudinal Associations

No significant association of A6MCT revolutions over time compared to other variables was seen. Total revolutions exhibit a trending correlation with time from baseline (*p* = 0.062). One outlier in the strong sitter group who combined an intense home exercise program with treatment drove this trending association. Consistent to reported real-world data in this patient population, all COAs were quite stable over a one year period with no significant difference (Table 5) [46,47].

## 4. Discussion

Existing literature and published data showing minimal detectable change for current clinical outcomes in SMA is based on research performed by highly experienced clinicians. However, specialized training is required for the administration and scoring of these outcomes (Clinician Rated Outcomes (CLINROs)), which can lead to variability in test results among clinicians with varying expertise. With the availability of disease modifying therapies (DMTs) and complementary treatments targeting downstream factors for therapeutic intervention, the use of Performance-based Outcome Testing (PerfO) becomes more relevant and aligned with these advancements. PerfO assessments involve tasks performed by patients and administered by clinicians, offering more objective results than patient-reported outcomes (PROs). This mitigates recall-related limitations and floor/ceiling effects [48]. There are currently limited COAs for measuring muscular endurance in non-ambulatory individuals with SMA, particularly for the very weak population. However, validated SMA measures of fatigability (such as the ESBBT and ESNHPT) exist, but they require fine motor skills that may be limiting for individuals who have weak grip or hand strength. Studies have highlighted mitochondrial dysfunction in SMA that may result in decreased endurance and higher fatigability [49,50]. 

The moderate to strong correlations observed between A6MCT revolutions and RULM, ATEND, and EK2 suggest that these measures offer complementary outcomes. This synergy may contribute to a more comprehensive understanding of motor function in SMA. This is corroborated by a published systematic review and meta-analysis of associations between motor competence and physical activity, in which six studies that examined the association between motor competence and muscular endurance were reviewed. The pooled correlation coefficient was significant, positive, and moderate for overall competence (r  =  0.34) [51]. Treatments aimed at influencing muscle endurance may benefit from utilizing clinically meaningful COAs designed to quantify and evaluate endurance. The integration of COAs measuring endurance can offer valuable insights into the efficacy and impact of interventions targeted at enhancing this physiological attribute.

### 4.1. Feasibility

We demonstrated that the A6MCT is a feasible measure of sustained maximal-effort activity and endurance for very weak non-ambulatory individuals with SMA. The participants in our study not only tolerated the A6MCT well but also provided positive feedback, expressing a sense of enjoyment in undertaking an exercise test that was previously inaccessible to them. The administration of the test within a clinical setting proved practical, lasting approximately 10–15 min and aligning with the typical time constraints of a clinic visit. This suggests that the A6MCT holds promise as a valuable tool for clinicians seeking to assess and monitor the muscular endurance of non-ambulatory individuals with SMA. 

Due to diminished dexterity and insufficient finger and grip strength, a subset of our patients encountered challenges in executing tasks within the RULM and 9HPT. Therefore we provided assistance gloves for those who had weak grip; most individuals were able to successfully perform the test with the gloves. Only those with severe elbow flexion contractures > 90 degrees were not able to complete one revolution, prompting discontinuation (*n* = 2). Overall, the A6MCT was feasible for 95% of the individuals tested in this study with the use of assistance gloves.

### 4.2. Relevance to Clinical Outcomes: Convergent Validity

The A6MCT has formerly been validated as a measure of endurance in the DMD population by showing associations with the MFM, a motor COA [31]. Further reports have suggested the A6MCT be validated against additional functional tests feasible for the non-ambulatory population due to limitations in completion of the 6MWT, the closest correlate [33]. 

Accordingly, this study demonstrates convergent validity with motor COAs showing significant correlations with total revolutions of A6MCT to ATEND, RULM and EK2. Notably, greater upper extremity and wheelchair-based function (higher scores on the RULM and ATEND scales) corresponded to increased sustained activity or endurance (total revolutions). As expected, given ATEND’s design focus on a very weak population, the non-sitter group exhibited significant associations between ATEND scores and total revolutions, unlike the sitter group. We observed that three individuals in the sitter group (7.9% of our population) reached a ceiling effect with the RULM but were able to demonstrate continued improvements with the A6MCT.

Greater patient-reported functional ability (lower EK2 score) was associated with higher sustained activity or endurance, in line with EK2’s negative correlation with motor function (*p* < 0.001). Performance fatigability was not associated with other COAs or PROs. However, these tests were not intended to be used to measure fatigability. These tests were intended to measure functional motor abilities, which suggests that muscle endurance may exhibit a closer connection to motor ability than performance fatigability does (utilizing comparison of first to last minute). Other factors may also impact effort for this patient population, which is not familiar with sustained effort activities. Thus they may not be exerting their best first minute effort compared to the final minute. The absence of a correlation between perceived fatigue (FSS) and performance fatigability highlights the possible difference between these two metrics of fatigue, implying they measure distinct constructs of fatigue. Our findings are consistent with other studies of assessing performance fatigability and FSS [38,52,53] in patients with SMA. 

### 4.3. Relevance to Clinical Characteristics and Change over Time

Performance fatigability effectively discriminated between sitters and non-sitters, confirming divergent validity. However, total revolutions did not exhibit a significant difference between the sitter and non-sitter groups. Upon closer examination of the ranges, sitters’ performance fell into substantial range, from 58 to 492 revolutions, whereas non-sitters performed within a narrower range, between 47 and 277 revolutions. While there may not appear to be a noteworthy distinction in muscular endurance, the observed difference in performance fatigability underscores the need for future investigation within our dataset to validate criterion validity.

Certain individuals with lower functioning levels cycled below the minimum assisted threshold of 52 revolutions without active user engagement. Clinical observations revealed that participants increasingly relied on compensatory trunk movements and momentum. This reliance on trunk compensations may impose additional resistance to cycling. These adaptations might amplify the effort exerted during the test, as the cumulative revolutions may encompass contributions from both upper limb and trunk functionality. Despite revolutions below the threshold, these three individuals reported RPE of >8 at end of A6MCT. Future examination of CPET data may offer additional insights into exertion levels for individuals with weaker musculature, and biomechanical analysis may elucidate the occurrence of muscle fatigability concurrent with increased trunk compensations.

Consistent with real-world observations of older individuals, our data also shows stability in the validated SMA clinical outcomes over time including the A6MCT [14,46,54]. While the A6MCT demonstrates a trend toward significance in relation to change over time, it is noteworthy that this trend was largely influenced by an outlier within our cohort. This outlier stands out because this individual was the one participant in our study who rigorously followed a consistent and intensive exercise program involving both strength training and aerobic activities throughout the period of the study. This case underscores the importance of exploring the potential impact of exercise as a complementary treatment to DMTs. For many weaker individuals, this test presented an opportunity to quantify a dimension of endurance that could not be measured by conventional assessments, leading to a sense of empowerment and accomplishment. 

### 4.4. Limitations

This is an initial investigation of a novel approach to assess an endurance-based measure for non-ambulatory individuals with SMA. We investigated a heterogenous population as indicated by RULM score ranging from 3 to 37. The small sample size and heterogeneity impacted results, which were further affected by a few outliers. Subsequent studies employing the A6MCT on a larger cohort, spanning from non-sitters to sitters and including weak walkers in their baseline functional abilities, would enhance the results presented here and refine the significance of the findings. 

Even though we assessed a wide range of individuals, a noteworthy observation is that there was no ceiling effect with the A6MCT. Contractures created a small floor effect limited by a small percentage of those who did not have the range of motion needed to complete a full revolution on the A6MCT. Exploring alternative methods of measuring endurance tailored to the limited range of motion in this population would be beneficial.

Nevertheless, the current methods have their limitations. The revolutions data from A6MCT did not exhibit a normal distribution and displayed left skewness. Although log-transforming the data could have mitigated these issues by equalizing effects and homogenizing the data, it would have compromised our ability to interpret the findings. To enhance our comprehension of the influence of data skewness on our analysis, we employed bootstrapping techniques and found no discernible difference in statistical significance. A more extensive sample size could effectively mitigate these potential risks.

Given the small, heterogenous, and non-normal distribution of our population, it is important to reflect on trending values rather than focusing only on statistical significance. The trending significance within the longitudinal Linear Mixed Model of total revolutions with function, SMN2 copy numbers, and disease duration should be noted and recommended for larger studies (Table 5). 

### 4.5. Future Directions

Subsequent investigations will prioritize the enhancement of psychometric attributes of the A6MCT, particularly concerning test-retest reliability and the establishment of the minimal detectable change and clinically important difference. Additionally, future research will explore a wider range of neuromuscular populations within a longitudinal study framework, including weak ambulatory individuals and a group that falls between sitters and walkers, as we suspect this group may exhibit a distinct trajectory of change [55,56]. Nevertheless, the two functional groups in our study continued to emerge as significant modifiers of change in both EK2 and performance fatigability.

This approach aims to foster a deeper comprehension of temporal transformations relative to well-established COAs, with a goal of establishing performance metrics of stamina, strength, and endurance to complement current motor measures. Future studies focusing on electromyography, near-infrared spectroscopy (NIRS), cardiopulmonary exercise testing (CPET), and exertion levels may provide enhanced differentiation between muscle fatigability and central or peripheral fatigue [55] by evaluating factors such as the rate of perceived exertion (RPE), metabolic intensity through respiratory gas analysis, hemodynamic parameters, and fatigue physiology of oxygenation in crucial upper extremity muscle groups. 

## 5. Conclusions

Current COAs require clinical expertise to accurately and reliably determine clinician-rated scales. Our data suggests that the Assisted 6-Minute Cycling Test can serve as a useful tool in clinical and research settings for evaluating endurance in non-ambulatory individuals with SMA. The metrics derived from A6MCT, such as total revolutions and percent fatigue, demonstrate the potential to detect change over time with minimal floor/ceiling effects. These metrics also offer an additional perspective on muscular endurance, complementing existing validated clinician-rated motor outcome measures. Such insights may contribute to a more comprehensive understanding of treatment responses and aid in the development of exercise recommendations and complementary metabolic and functional treatments.

## Figures and Tables

**Table 1:** Collected Variables and Outcomes.

Clinical Characteristics	Motor Function Outcomes	Patient-Reported Outcomes
Functional Status *Non-sitter*, *Sitter*	A6MCT *Total Revolutions*, *Percent Fatigue*	OMNI RPE ^3^ *Reported Minute 1 and Minute 6*
Age	RULM ^1^	FSS ^4^
*SMN2* Copy Number	ATEND ^2^	EK2 ^5^
Disease Duration		
Treatment Duration		

^1^ Revised Upper Limb Module (RULM), scores represent total from best side; ^2^ Adapted Test of Neuromuscular Disease (ATEND), scores represent total from best side; ^3^ Rate of Perceived Exertion (RPE), scores represent a 1–10 scale; ^4^ Fatigue Severity Scale (FSS), scores represent average; ^5^ Egen Klassifikation Version 2 (EK2), scores represent total.

**Table 2:** Descriptive Statistics of Clinical Characteristics.

	Overall (N = 38)
**Baseline Age (y)**	
Mean (SD)	30.3 (14.1)
Median [Min, Max]	27.4 [5.90, 74.1]
**Sex**	
Female	16 (42.1%)
Male	22 (57.9%)
**Baseline Disease Duration (y)**	
Mean (SD)	28.1 (14.6)
Median [Min, Max]	26.7 [0.110, 69.8]
**Baseline Treatment Duration (y)**	
Mean (SD)	3.17 (2.40)
Median [Min, Max]	3.01 [−0.444, 10.3]
**Baseline Treatment**	
Nusinersen	21 (55.3%)
Risdiplam	17 (44.7%)
**Function Group**	
Non-sitter	25 (65.8%)
Sitter	13 (34.2%)
**Type**	
Type 2	19 (50.0%)
Type 3	19 (50.0%)
*SMN2* Copy Number *	
Unknown ^†^	1 (2.6%)
2 SMN2	1 (2.6%)
3 SMN2	26 (68.4%)
≥4 SMN2	10 (26.3%)
**Gloves**	
Required	13 (34.2%)
Not Required	25 (65.8%)

y = years, * Note that SMN2 copy numbers may reflect “greater or equal to” the number shown, as some clinical labs reported “at least 2” or “at least 3” copies. ^†^ 1 patient had 1 SMN2 copy and a compound heterozygous point mutation on the single SMN1 allele.

**Table 3:** Descriptive Statistics of Baseline Outcomes.

	Non-Sitter (N = 25)	Sitter (N = 13)	Overall (N = 38)
**A6MCT: Total Revolutions**			
Mean (SD)	129 (77.0)	204 (180)	155 (125)
Median [Min, Max]	97.0 [47.0, 277]	93.0 [58.0, 492]	93.5 [47.0, 492]
**A6MCT: Percent Fatigue**			
Mean (SD)	13.9 (19.3)	−10.9 (27.0)	5.43 (24.9)
Median [Min, Max]	11.1 [−14.3, 48.5]	0 [−66.1, 33.8]	2.78 [−66.1, 48.5]
**RULM Best Side**			
Mean (SD)	15.8 (8.87)	25.6 (11.7)	19.5 (10.9)
Median [Min, Max]	17.0 [3.00, 37.0]	28.0 [5.00, 37.0]	18.5 [3.00, 37.0]
Missing	10 (40.0%)	4 (30.8%)	14 (36.8%)
**ATEND Best Side**			
Mean (SD)	26.4 (8.36)	30.3 (5.01)	27.7 (7.50)
Median [Min, Max]	26.5 [14.0, 41.0]	30.0 [24.0, 38.0]	28.0 [14.0, 41.0]
Missing	13 (52.0%)	7 (53.8%)	20 (52.6%)
**ATEND Upper Extremity Score**			
1	1 (4.3%)	0 (0%)	1 (2.6%)
2	1 (4.3%)	0 (0%)	1 (2.6%)
3	7 (30.4%)	3 (20.0%)	10 (26.3%)
4	5 (17.4%)	1 (6.7%)	6 (15.8%)
5	0 (0.0%)	3 (20.0%)	3 (7.9%)
6	0 (0.0%)	5 (33.3%)	5 (13.2%)
Missing	9 (39.1%)	3 (20.0%)	12 (31.6%)
**EK2 Total**			
Mean (SD)	15.5 (6.45)	15.6 (3.85)	15.5 (5.81)
Median [Min, Max]	15.0 [6.00, 28.0]	15.0 [11.0, 20.0]	15.0 [6.00, 28.0]
Missing	10 (40.0%)	8 (61.5%)	18 (47.4%)
**FSS Average**			
Mean (SD)	3.68 (1.38)	3.27 (1.68)	3.54 (1.46)
Median [Min, Max]	3.50 [1.57, 6.86]	2.86 [1.43, 5.71]	3.43 [1.43, 6.86]
Missing	11 (44.0%)	6 (46.2%)	17 (44.7%)
**Minute 6 RPE**			
Mean (SD)	6.67 (2.01)	6.70 (2.36)	6.68 (2.08)
Median [Min, Max]	6.00 [3.00, 10.0]	6.50 [2.00, 10.0]	6.00 [2.00, 10.0]
Missing	1 (4.0%)	3 (23.1%)	4 (10.5%)
**Minute 6 RPE Above 7**			
No	16 (64.0%)	10 (76.9%)	26 (68.4%)
Yes	9 (36.0%)	3 (23.1%)	12 (31.6%)

**Table 4:** Correlation Matrix of Baseline Data.

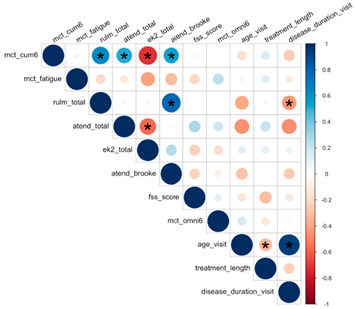
A * sign indicates *p*-value < 0.05
Gradient color of a circle indicates both magnitude and direction of correlation
The size of a circle indicates the magnitude of correlation
Magnitude of correlation ≠ significance of correlation

**Table 5:** Longitudinal Analysis for All Outcomes: Linear Mixed Model.

	A6MCT		A6MCT (No Outlier)		Fatigue %		RULM Total		ATEND Total		EK2 Total		ATEND Brooke		FSS Mean		Minute 6 RPE	
	Coefficient	*p* Value	Coefficient	*p* Value	Coefficient	*p* Value	Coefficient	*p* Value	Coefficient	*p* Value	Coefficient	*p* Value	Coefficient	*p* Value	Coefficient	*p* Value	Coefficient	*p* Value
**Intercept**	80.96	0.56	57.83	0.655	−4.36	0.853	29.94	0.012	19.2	0	23.82	0	3.88	0	3.4	0.038	4.9	0.029
**Time of Visit (yrs)**	29.79	0.062	−5.1	0.614	4.71	0.1	−1.16	0.372	−0.03	0.937	0.88	0.309	−0.2	0.277	−0.33	0.091	0.06	0.833
**Age**	2.84	0.629	3.28	0.506	0.59	0.502	0.01	0.963	1.92	0.186	0.43	0.146	0	0.993	−0.09	0.096	0.14	0.173
**Sitter**	94.02	0.079	72.13	0.121	−18.72	0.023	7.26	0.063	5.32	0.09	0.93	0.841	1.16	0.008	−1.15	0.084	0.51	0.554
***SMN*2 = 2**	304.19	0.115	279.61	0.116	40.99	0.159											1.41	0.649
***SMN*2 = 3**	70.1	0.585	82.55	0.507	21.69	0.262	−1.34	0.815	13.97	0.022	−17.28	0.008	−0.21	0.859	0.93	0.483	0.31	0.893
***SMN*2 = 4**	138.08	0.325	134.79	0.359	17.6	0.388	1.31	0.895	13.5	0.046	−20.19	0.005	0.09	0.903	2.46	0.111	0.65	0.771
**Treatment Duration**	5.04	0.596	8.88	0.315	0.26	0.872	0.09	0.843	0.26	0.771	0.77	0.245	0	0.987	−0.11	0.37	0.15	0.421
**Disease Duration**	−5.57	0.242	−5.34	0.1999	−0.82	0.298	−0.33	0.322	−2.23	0.125	−0.29	0.273	−0.02	0.569	0.08	0.108	−0.11	0.203

## Data Availability

The datasets generated and/or analyzed during the current study are not publicly available due to data containing private health information. Consent was obtained to provide cumulative anonymized data only. A minimal dataset is available from the corresponding author upon reasonable request.
